# The Complex Media Effects on Civic Participation Intention Amid COVID-19 Pandemic: Empirical Evidence from Wuhan College Students

**DOI:** 10.3390/ijerph191711140

**Published:** 2022-09-05

**Authors:** Xueyan Li, Ping Fu, Min Li

**Affiliations:** 1 School of Sociology, Central China Normal University, Wuhan 430079, China; 2 College of Marxism, Huazhong Agricultural University, Wuhan 430070, China

**Keywords:** media effect, media use, pandemic-relevant beliefs, civic participation intention, college students

## Abstract

In the context of the COVID-19 pandemic, media exposure is crucial to motivate public action for the combat with COVID-19 pandemic. However, media effects on civic participation intention are understudied. This study applied the Differential Susceptibility to Media effects Model (DSMM) to explore the relations among Wuhan college students’ media use, their pandemic-relevant beliefs, and civic participation intention, with a focus on the possible mediation of pandemic-relevant beliefs. Data of 4355 students from a large-scale cross-sectional survey were analyzed. Results show that traditional media use and online media interaction both directly and indirectly affect civic participation intention via pandemic-relevant beliefs. Pandemic-relevant beliefs distort the relations that direct and indirect effects of new media use on civic participation intention are significant but in opposite directions. The influence of pandemic news on civic participation intention is entirely mediated by pandemic-relevant beliefs. To conclude, during pandemic, the role of traditional media use is unreplaceable in its direct and indirect impact on civic participation intention. Pandemic-relevant beliefs play as a distorter variable. The balance between overexposure and insufficiency of pandemic-relevant news is vital. Online media interaction, as a main trait of new media use, plays a crucial role in civic participation intention, directly and indirectly.

## 1. Introduction

The COVID-19 pandemic has profound impact on public health and social lives. The consequences of the COVID-19 pandemic cannot be fully studied and understood without focusing on the complex effects of the mass media. In the context of the COVID-19 pandemic, the use of media, including traditional and new media, is the main approach for people to communicate when they are confined in their limited home space. Pandemic news floods in every day, occupying people’s attention and straining their nerves [1,2,3]. The psychological consequences of social media exposure amid the COVID-19 pandemic [1,2,3] are well studied; however, the media effects on civic participation intention are understudied during pandemics.

Civic participation is essential for a crisis like the COVID-19 pandemic, because compliance with health-protective guidelines and preventive measures dictated by the government is of great importance to restrain the spread of a pandemic. Civic participation intention, thus, is an indicator for whether people can be mobilized in the war towards the pandemic, and should be taken into account. The conceptualization of civic participation intention was inspired by the concept of community participation [4], which refers to a narrow concept such as voting in the earlier work [5], and then a broad concept including a wide range of activities in the later research, as defined by Zimmerman and Rappaport [6] (p. 726) as “any organized activities in which the individual participates without pay in order to achieve a common goal”. Civic participation intention in the present study was defined as the willingness to understand social policy, fulfill social responsibility and participate in social activities. Specifically, for college students, civic participation intention was operationalized as the interest in understanding national policy and public health administration and policy, the sense of social responsibility, the willingness of participating in anti-pandemic activities, and the confidence in one’s values for society.

In a severe global crisis like the COVID-19 pandemic, media is universally used as tools for providing authoritative information, allocating resources, mobilizing the community, and connecting isolated individuals [2,7]. The path from media exposure to civic participation and public action response is essential to combat the COVID-19 pandemic. Meanwhile, the COVID-19 pandemic has been an ideal breeding ground for online misinformation and conspiracy theories [8]. Due to the restrictions like home isolation and travel constraints, people are forced to stay at home, often idle, anxious, and hungry for information. The resulting spike in COVID-19 information increases the risk of exposure to misinformation and conspiracy theories. The consequences of ill-informed public have real-world implications in the form of low compliance with public health recommendations and directives [9,10]. How the path works from the media use to civic participation intention is an essential question to answer, and such answer, to some extent, determines the effectiveness and efficiency of the combat with COVID-19 pandemic. Therefore, to explore such a path from media use to civic participation intention is in urgent need.

Such a path can be complex because the COVID-19 pandemic is both the context in which media effects take place, and the content which occupies most attention of people in media use. Furthermore, the complexity of media effects resides in the divide between traditional and new media. New media (i.e., social media) is often blamed for the ongoing “deluge of conflicting information, misinformation and manipulated information” which, as some researchers argued, should be acknowledged as a global public health risk [9,11,12]. Unlike new media, traditional media is held in higher regard on its information quality and content regulation, but loses its appeal toward the young generation in its lack of social interaction.

In addition, the path from media use to civic participation intention can be various, and one possible mediator is the pandemic-relevant beliefs shaped by the process of media use. In the context of the COVID-19 pandemic, the effect of social media was often tagged with the shadow of conspiracy theories; in other words, the negative side of media effect captured much attention, while the bigger picture was often neglected, in which positive beliefs were transmitted via media for the public to uphold and inspire public active action in coping with the pandemic. Recent research mainly focused on the negative beliefs such as conspiracy theories [13], rather the positive beliefs. Beliefs in COVID-19 conspiracy theories negatively predict pandemic-related preventive and health-protective behavior, which in turn could severely undermine success in combating the COVID-19 pandemic [8,9,10]. However, Connolly et al. [14] noted that conspiracy theories are a marker of institutional distrust. It is the positive beliefs such as trust in the government’s anti-pandemic endeavors that are integral to motivating people’s positive actions. An empirical study [15] confirmed that trust in government officials has a significant and positive effect on compliance with COVID-19 guidelines and preventive measures. In view of this, in the present study, pandemic-relevant beliefs were defined as beliefs towards state institution, government ideal, individual identity, national unity, and effectiveness and contribution of anti-pandemic endeavors. Pandemic-relevant beliefs reflected individuals’ perceptions of nation and government action and reaction towards COVID-19. Such a definition tried to grasp the positive side of the media effects. In this sense, pandemic-relevant beliefs were opposite to conspiracy theories, and should gain more intention in studies of media effects.

Amid the outbreak of COVID-19 in December 2019 in Wuhan, Wuhan college students were a unique group, since they went home on winter holidays when the semester ended in late December 2019 and early January 2020, and therefore, as potential virus carriers, had a clear migration route from Wuhan to their hometown which could be traced for virus spreading control measures. Their compliance with college guidance and government guidelines was crucial for curbing the spread of the virus. Social media, such as QQ and WeChat, is widely used for pandemic-relevant communication in colleges. Fan, Li et al.’s study [16] estimated that the COVID-19 infection rate of Wuhan college students was 1.61‰ and found that infection primarily happened off campus or in their hometown. Such a relatively lower infection rate may be partly due to their compliance with public health recommendations and directives. Furthermore, young people’s active participation in anti-pandemic action reflects the achievements of the Chinese government in COVID-19 prevention and control.

### 1.1. Research Objectives

In view of this, the present study intended to investigate the relationship among Wuhan college students’ media use, their pandemic-relevant beliefs, and civic participation intention with the pandemic, with particular focus on the possible mediation of pandemic-relevant beliefs between media use and civic participation intention.

### 1.2. Theoretical Framework

This study was not only a study on media effects in the context of the COVID-19 pandemic, but also particularly chose individual’s civic participation to see how media use affects such civic participation intention, directly or indirectly. Therefore, a framework, the Differential Susceptibility to Media effects Model (DSMM), proposed by Valkenburg and Peter [17,18,19] was applied for the present investigation. The DSMM model makes four propositions: (1) Media effects rely on three kinds of differential susceptibility variables. (2) The relations between media use and effects are mediated by three media response states. (3) The differential susceptibility variables act as both predictors and moderators. (4) Media effects are transactional.

Besides its use for explaining individual differences in susceptibility to media effects as stated in proposition 1 and proposition 3, DSMM can be used for explaining the process of media influence as in proposition 2. The mediator between media use and outcomes can be either media response states, i.e., “the mental and physiological processes that occurs during media use” (p. 223), or mediating media effects that “provide the underlying mechanism of (causal route to) second-order media effects” (p. 224). The former usually occur during media use, while the latter can start media use but last beyond the media use situation.

**Figure 1:**
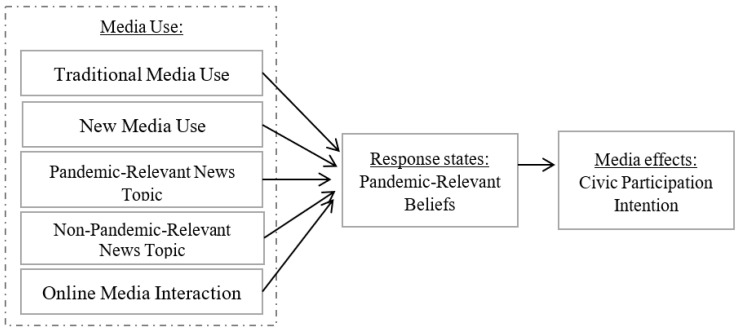
Theoretical framework of the complex media effects on civic participation intention.

As shown in Figure 1, the present study mainly used proposition 2, that is, response states mediate the relation between media use and effects. Specifically, media use was operationalized as five variables of three aspects, including media type (traditional media use and new media use), media content (pandemic media content and non-pandemic media content), and media online interaction. Response states were operationalized as pandemic-relevant beliefs, which were mostly cognitive. Media effects were operationalized as civic participation intention. The main purpose was to empirically establish the mediation of pandemic-relevant beliefs between media use and media civic participation. Accordingly, the present study proposed that Wuhan college students who used traditional media or new media, formulated specific beliefs about the pandemic and pandemic-relevant issues, such as anti-pandemic endeavors and faith in government. Such beliefs can further trigger or lead to civic participation intention with pandemic-relevant actions.

Around the relations between media use and civic participation intention, one string of studies focuses on the concept of conspiracy beliefs (i.e., the conspiracy theory of society), which assume public events are secretly controlled and orchestrated by powerful entities [20]. New media (i.e., social media), rather than traditional media, has long been recognized as a major disseminator of health misinformation. Therefore, the use of new media increases the risk of exposure to conspiracy beliefs, which further affects individual’s choice of action. The underlying logic here is to assume beliefs as mediators between media use and civic participation. However, such mediating effects are rarely taken into account and empirically tested in the context of the COVID-19 pandemic. Some research reported a positive relationship between the use of social media and COVID-19 conspiracy beliefs, a negative association between conspiracy beliefs and health-protective behaviors, and a negative relationship between the use of social media and COVID-19 health-protective behaviors [8,9]. The negative association between conspiracy beliefs related to other health matters such as vaccination and health-protective behaviors are also empirically supported in previous studies [21,22,23]. On the basis of these studies, it seemed reasonable to infer the potential mediating effect of beliefs between the use of social media and behaviors. However, the possible mediating role of conspiracy beliefs was left empirically unexplored. More importantly, in these studies, beliefs as underlying mediators were simplified only as conspiracy beliefs, while positive beliefs which actually represented the mainstream beliefs in the COVID-19 era were neglected.

The outbreak of COVID-19 characterized by ignorance and high uncertainty, provides a breeding ground for conspiracy beliefs. COVID-19 misinformation exposure was associated with misinformation beliefs, while misinformation beliefs were associated with fewer preventive behaviors [9,10]. Such a path seems reasonable and evidence-based, but what about the role of positive beliefs? Do positive beliefs act like conspiracy beliefs? Positive beliefs, i.e., pandemic-relevant beliefs in this study, refer to beliefs towards state institution, government ideal, individual identity, national unity, and effectiveness and contribution of anti-pandemic endeavors. Enlighted by the previous studies regarding conspiracy beliefs, as shown in Figure 1, this study hypothesized that

**Hypothesis** **1** **(H1).** *Media use (including traditional media use, new media use, pandemic-relevant news, non-pandemic news, online media interaction) relates to pandemic-relevant beliefs. Specifically, the use of new media increases the risk to exposure to conspiracy beliefs*.

**Hypothesis** **2** **(H2).** *Pandemic-relevant beliefs relate to civic participation intention with pandemic*.

**Hypothesis** **3** **(H3).** *Media use affects civic participation intention directly, and indirectly* via *the mediation of pandemic-relevant beliefs*.

## 2. Methods

### 2.1. Study Design and Sampling

Unlike most previous studies on media use during the COVID-19 pandemic [2,24,25] which used social media platforms such as Weibo or WeChat for data collection and usually only reached a sample size of a few hundred, this study used data from a large-scale cross-sectional survey conducted from 26 to 29 April 2020, with a multistage random sampling scheme, COVID-19 Impact Survey of Faculty and Students of Wuhan Universities and Colleges (CFSW). The detailed information on design, participants, and procedure can be referred to in Li, et al. [26] and Fan, et al. [16,27]. In brief, out of all 83 universities and colleges located in Wuhan City, Hubei Province, the researcher selected 2 public universities affiliated with the state, 5 public universities affiliated with the province, 2 public colleges affiliated with the province, and 4 private universities and colleges affiliated with the province. Within each sampled university and college, 30% of the schools and departments were systematically selected. With each sampled school and department, students were randomly selected according to their program and grade. 4355 students participated in the present study.

### 2.2. Measurements

#### 2.2.1. Media Use

Media use was measured in three aspects. The first aspect is media type, and participants were asked the following question: “What is your main way to get access to information during the Wuhan lockdown period (multiple choice)?” (1) domestic radio, television, and other traditional media; (2) domestic new media; (3) foreign traditional media; (4) foreign new media. Four options were recoded into two variables. Option (1) and (3) were combined and recoded into the variable traditional media use (value 0 and 1); option (2) and (4) new media use (value 0 and 1).

The second aspect is media content, indicated by the news topics, and participants were asked the following two questions: “What are the domestic news topics you have been most interested in (multiple choice)?” (1) pandemic status and development trend; (2) pandemic response measures; (3) domestic economic situation; (4) domestic political situation; (5) social situation; (6) emergency matters; (7) doctor-patient relationship; (8) others. “What are the overseas news topics you have been most interested in (multiple choice)?” (1) overseas epidemic situation and development trend; (2) overseas media propaganda and public opinion direction; (3) overseas media’s interpretation of China’s anti-COVID measures; (4) international economic environment and influence; (5) international political situation; (6) international relations; (7) others. Options (1) and (2) in the first question and options (1) and (3) in the second question were combined and recoded into the variable pandemic media content (values 0 and 1), and all the remaining options of the two questions were non-pandemic media content (value 0 and 1).

The third aspect is media online interaction, and participants were asked the following question: “How have you participated in online interaction (multiple choice)?” (1) posting a “like”; (2) posting and commenting; (3) knowledge sharing (including reposting); (4) initiating online discussion and promotion; (5) being interviewed by media; (6) participating in online training and lectures; (7) others. All options were combined and recoded into the variable media online interaction (values 0 and 1).

#### 2.2.2. Pandemic-Relevant Beliefs

Nine items were designed for the particular use to measure pandemic-relevant beliefs in the present study, including beliefs towards state institution, government ideal, individual identity, national unity, and effectiveness and contribution of China’s anti-pandemic endeavors. Pandemic-relevant beliefs reflect students’ perceptions of nation and government action and reaction towards COVID-19 during the Wuhan lockdown period. Items are presented in Table 1. The question was “After this COVID-19 epidemic, how much do you agree with the following statements?” Participants were asked to rate all the items from lowest (0) to highest (4), which were then recoded as 1 (lowest) to 5 (highest) for further analysis. The Cronbach alpha coefficient was 0.963. An exploratory factor analysis revealed one factor, accounting for 78.58% variance. The high variance accounted for by only one factor states a high correlation among these items, manifesting good construct validity. These beliefs typically occur during media use, but also probably last afterward. The average score of all nine items measuring pandemic-relevant beliefs was used for further analysis.

#### 2.2.3. Civic Participation Intention

Five items were designed for the particular use to measure civic participation intention in the present study, including interest in understanding national policy and public health administration and policy, the sense of social responsibility, willingness of participating in anti-pandemic activities and confidence in one’s professional values for society. The question was “After this COVID-19 epidemic, how much do you agree with the following statements?” Items are presented in Table 1. Participants were asked to rate all the items from lowest (0) to highest (4), which were then recoded as 1 (lowest) to 5 (highest) for further analysis. The Cronbach alpha coefficient was 0.841. An exploratory factor analysis revealed one factor, accounting for 68.03% variance. The high variance accounted by the only one factor states high correlation among these items, manifesting good construct validity. The average score of all five items measuring civic participation intention was used for further analysis.

### 2.3. Statistical Analysis

#### 2.3.1. Mediation Analysis

The PROCESS macro developed by Hayes [28] was used for mediation analysis. The intention of mediation analysis is to explore the possible process in which the independent variable X influence the dependent variable Y, and determine whether the relation between X and Y is partially or totally attributed to the mediator variable M. Therefore, a mediator variable M reflects how an independent variable X affects the dependent variable Y through the mediation of M. In this study, the independent variable X is media use, including traditional media use, new media use, pandemic news, non-pandemic news, and online media interaction; the dependent variable is pandemic civic participation intention, and the mediator variable is pandemic-relevant beliefs.

The classic method for determining the mediation effect is the Baron and Kenny [29] causal steps model through stepwise regression combined with the Sobel test. However, this method requires three significant correlations among X, M, and Y before examining the mediation effect. MacKinnon’s [30] argued that mediation can be found when the correlation between X and Y is not significant. In such case, M can play as a distorter variable in which the direction of relation between X and Y can emerge when M is adjusted for. In this study, in line with MacKinnon [31], the possible distortion effect is explored when the correlation between X and Y is not significant.

#### 2.3.2. Control Variables

A range of demographic variables of students were considered as control variables in the present study, including gender (male and female), age cohort (born before 1995, born between 1995 and 2000, and born after 2000), university category (public university affiliated with the state/province, public college affiliated with the province, and private university and college affiliated with the province), program (junior bachelor, bachelor, master’s and doctoral), and living place during pandemic (Wuhan, other places in Hubei, and other provinces). Since all the control variables were categorical, a series of dummy variables were recoded for further analysis.

## 3. Results

### 3.1. Overview

The mean and standard deviation of variables are shown in Table 2. During the Wuhan lockdown period, 85% of Wuhan college students used traditional media to obtain information, while 94% used new media. The high proportion of new media use is expected in such a new media era; the high proportion of traditional media use is not surprising as well, and is mainly attributed to the circumstance in which college students were required to isolate at home, and thus passively got access to traditional media such as TV. Students were interested in both pandemic news and non-pandemic news, and the former (98%) had a slightly higher proportion than the latter (95%). Such a high proportion of 98% indicates that it is almost not possible to escape from the flooding news coverage of COVID-19 during the pandemic time. The landscape of media is undoubtedly dominated by COVID-19 pandemic news. Moreover, 60% of college students participated in online interactions such as sharing or reposting knowledge, and commenting on others’ posts. Such online interaction mainly happened on social media platforms such as WeChat and Weibo, and accounted for a big proportion of college students’ social life during the lockdown period.

In order to examine the impact of intensive media use amid the COVID-19 pandemic, and more importantly, to determine the possible mediation effect of pandemic-relevant beliefs between media use and civic participation intention, the correlation analyses were conducted and shown in Table 2. First, the correlation between X and Y was examined, and the results showed that civic participation intention is significantly correlated with traditional media use (r = 0.11, *p* *<* 0.001), pandemic news (r = 0.06, *p* *<* 0.001), and online media interaction (r = 0.08, *p* *<* 0.001), but not correlated with new media use and non-pandemic news. Second, the correlation between X and M showed that pandemic-relevant beliefs are significantly correlated with traditional media use (r = 0.04, *p* = 0.008), new media use (r = 0.05, *p* < 0.001), pandemic news (r = 0.10, *p* < 0.001) and online media interaction (r = 0.07, *p* < 0.001), but not with non-pandemic news. Third, the correlation between M and Y showed that pandemic-relevant beliefs are significantly related to civic participation intention (r = 0.63, *p* < 0.001).

Three independent variables (i.e., traditional media use, pandemic news, and online media interaction) satisfied the requirements by Baron and Kenny (1986) causal steps model, and one independent variable, i.e., new media use satisfied the requirement by MacKinnon [30]. The independent variable of non-pandemic news did not satisfy any of the requirements, and was therefore excluded from the further mediation analysis.

### 3.2. Media Use and Civic Participation Intention

In step 1, the regression of media use on civic participation intention shows that traditional media use (β = 0.111, *p* < 0.001), pandemic news (β = 0.057, *p* < 0.001), and online media interaction (β = 0.079, *p* < 0.001) significantly affect civic participation intention. Hypothesis H1 was supported.

### 3.3. Media Use and Pandemic-Relevant Beliefs

In step 2, the regression of media use on pandemic-relevant beliefs shows that traditional media use (β = 0.040, *p* = 0.008), new media use (β = 0.051, *p* < 0.001), pandemic news (β = 0.096, *p* < 0.001), and online media interaction (β = 0.066, *p* < 0.001) significantly affect pandemic-relevant beliefs.

Moreover, in step 3, the regression of pandemic-relevant beliefs on civic participation intention is significant (β = 0.634, *p* < 0.001). Hypothesis H2 was confirmed.

### 3.4. The Mediation of Pandemic-Relevant Beliefs in Relation between Media Use and Civic Participation Intention

In step 4, when the impact of pandemic-relevant beliefs was adjusted for in the regression analysis of civic participation intention on media use, the regression coefficient of traditional media use (β = 0.086, *p* < 0.001) and online media interaction (β = 0.037, *p* = 0.002) remains significant but decreases in magnitude; the coefficient of new media use emerges to be significant (β = −0.026, *p* = 0.03); the coefficient of pandemic news is no longer significant (β = −0.004, *p* > 0.05). Results from the four steps of mediation analysis are shown in Table 3. Furthermore, when the control variables (gender, age cohort, university category, program, and living place during the pandemic) were taken into consideration, the results remained robust and thus, were not presented here.

In order to examine the direct and indirect effects between media use and civic participation intention, a 95% confidence interval with bootstrapping method was computed as in Table 4. In the path from traditional media use to civic participation intention, both the direct effect (CI = 0.1184, 0.2045) and indirect effect via pandemic-relevant beliefs (CI = 0.0105, 0.0877) are significant, so is the total effect (CI = 0.1538, 0.2649). Therefore, the partial mediation is established, and the impact of traditional media use on civic participation intention is partially mediated through pandemic-relevant beliefs.

In the path from new media use to civic participation intention, as noted earlier, the correlation between these two variables is not significant. As in line with this result, the total effect of new media use on civic participation intention is not significant (CI = −0.0647, 0.1041). However, when the variable of pandemic-relevant beliefs is adjusted for, the effect of new media emerges to be significant (β = −0.026, *p* < 0.001). Both indirect effect (CI = 0.0257, 0.1650) and direct effect (CI = −0.1377, −0.0071) emerges to be significant, but with opposite directions. Such a mediator variable is named a distorter variable by MacKinnon [30].

In the path from pandemic news to civic participation intention, the indirect effect through pandemic-relevant beliefs is significant (CI = 0.1349, 0.4172), but the direct effect (CI = −0.1211, 0.0829) is not. In other words, the influence of pandemic news on civic participation intention is entirely mediated by pandemic-relevant beliefs (CI = 0.1194, 3815), and the full mediation effect is established.

In the path from online media interaction to civic participation intention, both the direct effect (CI = 0.0192, 0.0829) and indirect effect via pandemic-relevant beliefs (CI = 0.0310, 0.0858) are significant, so is the total effect (CI = 0.0679, 0.1499). Therefore, the partial mediation is established.

In a word, Hypothesis H3 was confirmed for the variables of traditional media use, new media use, pandemic news, and online media interaction, and such mediation effects demonstrated three types, i.e., full mediation, partial mediation, and distortion effect. Hypothesis H3 was not held for the variable of non-pandemic news.

## 4. Discussion

### 4.1. Principal Findings

#### 4.1.1. The Temporary Re-Claim of Traditional Media Use

This study reports a high proportion of both traditional and new media use of Wuhan college students during the lockdown period. However, the high proportion of traditional media use may largely be attributed to the particular circumstance in which college students were required to stay at home as part of COVID-19 prevention measures and therefore got access to TV owned by almost every Chinese family. Meanwhile, according to a report released by the China Internet Network Information Center (CNNIC) [31], as of June 2021, China’s Internet users reached 1.011 billion, with an increase of 21.75 million from December 2020, and the Internet penetration rate reached 71.6%. In particular, the communication infrastructure in rural areas has been gradually improved, and the cost of using the Internet has been gradually reduced. More than 99% of administrative villages have access to optical fiber and 4G, realizing the “same Internet speed” in both rural and urban areas, which, to a large extent, ensures that rural college students can obtain information through traditional and new media after returning home.

The external physical discipline exerted on an individual’s body, the limited living space at home, and the few family members to interact with, all together change an individual’s routine, including their preference to media use. The proportion of new media use remains high. The traditional media once again, although may temporally, claim its territory in media use. However, once students go back to their normal college life, traditional media will then lose its power.

On the other side, it cannot be denied that traditional media still plays an influential role in life, especially during global crisis times like the COVID-19 pandemic. Chao, Xue, et al.’s study [2] found that compared with new media use, traditional media use is significantly associated with less negative affect, anxiety, and stress during the COVID-19 outbreak in China. The point that traditional media use may trigger less negative affect means a great deal in chaotic times such as the COVID-19 outbreak. The differential impact of traditional and new media use, therefore, calls for further attention here.

#### 4.1.2. The Undoubtedly Dominance of Pandemic News

One of the motives for this study is to look into the pervasive impact of the COVID-19 pandemic on the media landscape. As stated by Houston, Pfefferbaum, & Reyes [32] (p. 14) “when a major disaster occurs, coverage of the event typically floods the media landscape.” The almost full occupation of attention on pandemic news is the best demonstration for the pandemic narrative dominance. The concept of “infodemic” has been used to emphasize on the overflood of information, with the intention to examine its negative impact on individuals’ mental health [2,8,33]. Although the mental health consequence of infodemic is beyond the discussion of the present study, such a high volume of pandemic news on an individual’s beliefs and civic participation intention is important here.

#### 4.1.3. The Complex Mediation of Pandemic-Relevant Beliefs

In the present study, participants’ mean score of pandemic-relevant beliefs is high (mean = 4.77), showing a high endorsement of Wuhan college students on their positive beliefs towards state institution, government ideal, individual identity, national unity, and effectiveness and contribution of China’s anti-pandemic endeavors. This result is in line with recent studies. A large-scale survey found that Chinese citizens have an overall high level of satisfaction with their government’s performance during the COVID-19 pandemic and such satisfaction enhanced public support for the Chinese government [34]. Specifically, 47.6% of citizens’ trust in the national government remain the same, and 49.2% trust more. The evidence for such lasting or even increased trust can be found in other studies. World Values Survey [35] shows that Chinese citizens rate a score of 7.5 for their satisfaction with the national government on a scale ranging from 0 (extremely dissatisfied) to 10 (extremely satisfied). The present study adds evidence for the high satisfaction and positive beliefs of college students, who represent a large proportion of youth population.

Participants’ mean score of civic participation intention is also high (mean = 4.35), showing a high endorsement of the interest in understanding national policy and public health administration and policy, the sense of social responsibility, the willingness of participating in anti-pandemic activities, and confidence in one’s values for society.

As mentioned earlier, the integrative media effects research framework (DSMM) proposed by Valkenburg and Peter [17,18,19] mainly focuses on cognitive, emotional, and excitative media response states that mediate media effects, but lack of attention on behavioral effects of media. The present study not only considers the type, topic, and trait of media use, but also further extends the DSMM framework to individual behaviors. Media use in this study includes traditional media use, new media use, pandemic news, non-pandemic news, and online media interaction. The relations among media use, pandemic-relevant beliefs, and civic participation intention demonstrate complexity. Four types of mediation effects of pandemic-relevant beliefs are spotted: (1) partial mediation, pandemic-relevant beliefs partially mediate the effect of traditional media use on civic participation intention, and the effect of online media interaction on civic participation intention; (2) full mediation, pandemic-relevant beliefs entirely mediate the effect of pandemic news on civic participation intention; (3) distorter effect, pandemic-relevant beliefs act as a distorter variable, since the non-significant effect of new media use on civic participation intention becomes significant when pandemic-relevant beliefs are adjusted for in the analysis; (4) no mediation, both direct and indirect effects of pandemic-relevant beliefs between non-pandemic news and civic participation intention are not significant.

The findings that multiple types of mediation co-exist in terms of the relations between media use and civic participation intention provide strong evidence for the complexity of media effects amid the COVID-19 pandemic.

First, traditional media use and new media use exert its force on civic participation intention in different ways. Traditional media can both directly and indirectly affect civic participation intention, which demonstrates its important role during crisis times. The indirect effect could be channeled by the pandemic-relevant beliefs. Some research reported the differential impact of traditional and new forms of media coverage, such as terrorist attacks [36,37], hurricane disasters [38], and the COVID-19 pandemic on mental health [2], and compared with traditional media use, new media use triggers more negative affect, such as anxiety and stress [3,39,40]. The current findings of the differential impact of traditional and new media use seem to further expand such impact from mental state to behavioral civic participation intention.

Second, it seems unexpected and counterintuitive to see the non-significant correlation between new media use and civic participation intention. However, as MacKinnon [30] and other researchers [41,42,43] argued, the significant mediational process is possible even if the overall relation between independent and dependent variables is not significant; in other words, the overall significant relation is not a must requirement for mediation tests. The non-significant relation between new media use and civic participation intention becomes significant when the variable of pandemic-relevant beliefs is included in the analysis. In this case, the variable of pandemic-relevant beliefs plays as a distorter variable [44]. It seems intuitively difficult to understand a distorter variable. Taking the example from Rosenberg [44], suicide rates were higher for the married than the unmarried; however, when age was used for stratification, suicide rates were actually lower for the married than the unmarried for most age groups; age plays as a distorter variable, since only when age was included, the correct interpretation was revealed. Analogically, the inclusion of distorter variable pandemic-relevant beliefs changes the interpretation of the relation between new media use and civic participation intention and unveil the true relation between them. Besides, it is possible that some other variables channel the indirect effect between new media use and civic participation intention, which calls for further exploration.

Third, the variable of pandemic news was used to indicate the particular characteristics of the COVID-19 pandemic. As expected, the impact of pandemic news on civic participation intention was totally mediated by pandemic-relevant beliefs. The differentiation between traditional media use and new media use is to capture the type difference; while the pandemic news topic is to capture the content of media use. The finding of the total mediation implies that the clear and authoritative news coverage on traditional media stabilizes people’s moods and stimulates their positive beliefs, which then can mobilize their civic participation intention. Media use indeed affects the way that people view the world [45].

Fourth, the variable of online media interaction was used to represent the uniqueness of new media use, which is a lack in traditional media use. Online mediation interaction can both directly and indirectly affect civic participation intention. People use new media to access crowd-sourced information, interact with the public, and thus get more engaged with a new media environment. New media makes a huge impact on disaster and emergency communication [46]. However, new media was under blame for its lack of regulation in content quality and the proliferation of misinformation [8,9]. Meanwhile, the gradual replacement of new media for traditional media seems irreversible. Therefore, how to harness the power of new media to shape flattering attitudes and beliefs toward society, and then to actively involve public participation seems particularly important in success in coping with a global crisis such as the COVID-19 pandemic.

The very recent Munich Security Report 2021 describes China as ”an unperturbed country”, and such a view is mainly based on China’s performance amid the COVID-19 pandemic. The pandemic did not shake, rather even increase Chinese citizens’ trust in their government, which was not as some [47,48,49,50] predicted or believed. How did that happen? As stated by Hua & Shaw [33], a unique combination of strong governance, strict regulation, strong community vigilance, and citizen participation, and wise use of big data and digital technologies, were some of the key factors in China’s efforts to combat this virus. The findings of the present study seem to give away a glimpse of such a combination. In striking contrast, a study on a nationally representative sample of 1252 participants of a European country reported only 59.4% high trust in scientists and satisfaction in government, and low beliefs in conspiracy during the COVID-19 pandemic [13].

### 4.2. Limitations

Although our large-scale study provides evidence of the role of pandemic-relevant beliefs in the effect of media use on civic participation intention during the COVID-19 pandemic, there are limitations that need to be recognized. We used a multistage random sample, but the questionnaire was conducted online, which restricted the sample to those with online access. As a result, college students from disadvantaged areas with limited internet infrastructure were underrepresented, although they only occupied a limited proportion according to CNNIC. Additionally, because we relied on students’ self-reports of civic participation intention, we could not confirm that those reports reflected their actual behavior. Furthermore, latent profile analysis of students’ actual behavior will be interesting in future studies. Our results were also only applicable to situations in the time when our survey was conducted. The effects of media use may change along with the COVID-19 pandemic varies.

## 5. Conclusions

To conclude, this study finds:

1. During crisis times such as the COVID-19 pandemic, the role of traditional media use seems unreplaceable in its direct and indirect impact on civic participation intention, although new media is gradually replacing traditional media.
2. The influence of new media is more complex, and mediator variables must be taken into consideration to reveal a correct conclusion. Pandemic-relevant beliefs play as a distorter variable since it reveals the significant but opposite direct and indirect effect of new media on civic participation intention.
3. Pandemic-relevant news is in great needs, and the balance between overexposure and sufficient supply is always a tricky problem. However, the proper coverage can lead to wide civic participation intention.
4. Online media interaction, as a main trait of new media use, plays a crucial role in civic participation intention, directly and indirectly.

## Figures and Tables

**Table 1:** Measurement items.

Variables	Items
Media use	Traditional media use (Yes = 1, No = 0)
	New media use (Yes = 1, No = 0)
	Pandemic-relevant news topic (Yes = 1, No = 0)
	Non-pandemic-relevant news topic (Yes = 1, No = 0)
	Online media interaction (Yes = 1, No = 0)
Pandemic-relevant beliefs	1. Socialism with Chinese characteristics has great superiority.
	2. The Party Central Committee with General Secretary at the core always insists that the interests of the people are above all else.
	3. I am proud of being a Chinese.
	4. China has effectively controlled the spread of the epidemic in the short term, bought time for the world to fight the epidemic, and provided a Chinese model for the world to fight the epidemic.
	5. China provides support to countries or regions with severe epidemics, reflecting the responsibility of a responsible country.
	6. I am very moved to see that the people all over the country unite their efforts to support Wuhan.
	7. The Party and the country are very concerned about the people of Hubei and Wuhan.
	8. The closure of Wuhan is timely, necessary, and important.
	9. Overseas Chinese support China’s fight against the epidemic very well.
Civic participation intention	1. My interest in understanding national policies has increased.
	2. My understanding and knowledge of public health and policy has increased.
	3. I feel I can take on a certain amount of responsibility for my family and society.
	4. I am willing to participate in pandemic-related activities in the community (village).
	5. I feel my professional value has been brought into play.

**Table 2:** Descriptive statistics of media use, pandemic-relevant beliefs, and civic participation intention (*n* = 4355).

	M	SD	1	2	3	4	5	6
1 Traditional media use	0.85	0.36	1					
2 New media use	0.94	0.24	−0.11 ***	1				
3 Pandemic news	0.98	0.15	0.07 ***	0.04 *	1			
4 Non-pandemic news	0.95	0.22	0.11 ***	0.15 ***	−0.04 *	1		
5 Online media interaction	0.60	0.49	0.09 ***	0.08 ***	0.06 ***	0.08 ***	1	
6 Pandemic-relevant beliefs	4.77	0.52	0.04 **	0.05 ***	0.10 ***	0.03	0.07 ***	1
7 Civic participation intention	4.35	0.68	0.11 ***	0.01	0.06 ***	0.01	0.08 ***	0.63 ***

Note: * *p* < 0.05, ** *p* < 0.01, *** *p* < 0.001.

**Table 3:** The mediation of pandemic-relevant beliefs between media use and civic participation intention.

Step	IV	DV	Path	β	S.E	R^2^	F
Step 1	1 Traditional media use	Civic participation intention	c1	0.111 ***	0.028	0.012	54.556 ***
	2 New media use		c2	0.007	0.043	0.000	0.209
	3 Pandemic news		c3	0.057 ***	0.067	0.003	14.039 ***
	5 Online media interaction		c5	0.079 ***	0.021	0.006	27.072 ***
Step 2	1 Traditional media use	Pandemic-relevant beliefs	a1	0.040 **	0.022	0.002	7.099 **
	2 New media use		a2	0.051 ***	0.033	0.003	11.381 ***
	3 Pandemic news		a3	0.096 ***	0.052	0.009	40.673 ***
	5 Online media interaction		a5	0.066 ***	0.016	0.004	19.119 ***
Step 3	Pandemic-relevant beliefs	Civic participation intention	b	0.634 ***	0.015	0.402	2923.102 ***
Step 4	1 Traditional media use	Civic participation intention	c1′	0.086 ***	0.022	0.409	1506.475 ***
	2 New media use		c2′	−0.026 *	0.033	0.402	1465.163 ***
	3 Pandemic news		c3′	−0.004	0.052	0.402	1461.328 ***
	5 Online media interaction		c5′	0.037 **	0.016	0.403	1469.460 ***

Note: IV, independent variable; DV, dependent variable. * *p* < 0.05, ** *p* < 0.01, *** *p* < 0.001.

**Table 4:** The mediating effects of pandemic-relevant beliefs between media use and civic participation intention.

	Model	Product of Coefficients		Boot 95% CI	
		Point Estimate	BootSE	Lower	Higher
Traditional media use→civic participation intention	Total	0.2093	0.0283	0.1538	0.2649
	Direct	0.1615	0.0219	0.1184	0.2045
	Indirect	0.0479	0.0198	0.0105	0.0877
New media use→civic participation intention	Total	0.0197	0.0430	−0.0647	0.1041
	Direct	−0.0724	0.0333	−0.1377	−0.0071
	Indirect	0.0921	0.0351	0.0257	0.1650
Pandemic news→civic participation intention	Total	0.2505	0.0668	0.1194	0.3815
	Direct	−0.0191	0.0520	−0.1211	0.0829
	Indirect	0.2696	0.0708	0.1349	0.4172
Online media interaction→civic participation intention	Total	0.1089	0.0209	0.0679	0.1499
	Direct	0.0511	0.0163	0.0192	0.0829
	Indirect	0.0578	0.0138	0.0310	0.0858

## Data Availability

The data presented in this study are available on request from the corresponding author. The data are not publicly available due to privacy restrictions.
